# Accuracy of a Low-Cost 3D-Printed Wearable Goniometer for Measuring Wrist Motion

**DOI:** 10.3390/s21144799

**Published:** 2021-07-14

**Authors:** Calvin Young, Sarah DeDecker, Drew Anderson, Michele L. Oliver, Karen D. Gordon

**Affiliations:** School of Engineering, University of Guelph, Guelph, ON N1G 2W1, Canada; sdedecke@uoguelph.ca (S.D.); dander04@uoguelph.ca (D.A.); moliver@uoguelph.ca (M.L.O.); kgordon@uoguelph.ca (K.D.G.)

**Keywords:** wearable device, electromechanical goniometry, occupational biomechanics

## Abstract

Wrist motion provides an important metric for disease monitoring and occupational risk assessment. The collection of wrist kinematics in occupational or other real-world environments could augment traditional observational or video-analysis based assessment. We have developed a low-cost 3D printed wearable device, capable of being produced on consumer grade desktop 3D printers. Here we present a preliminary validation of the device against a gold standard optical motion capture system. Data were collected from 10 participants performing a static angle matching task while seated at a desk. The wearable device output was significantly correlated with the optical motion capture system yielding a coefficient of determination (R${}^{2}$) of 0.991 and 0.972 for flexion/extension (FE) and radial/ulnar deviation (RUD) respectively (*p* < 0.0001). Error was similarly low with a root mean squared error of 4.9° (FE) and 3.9° (RUD). Agreement between the two systems was quantified using Bland–Altman analysis, with bias and 95% limits of agreement of 3.1° ± 7.4° and −0.16° ± 7.7° for FE and RUD, respectively. These results compare favourably with current methods for occupational assessment, suggesting strong potential for field implementation.

## 1. Introduction

Wrist motion is a valuable metric in many fields, including orthopaedic surgery, hand and upper extremity rehabilitation and therapy, ergonomics, athletics, and other areas that relate to the performance of a task involving the hands and upper extremity. The wrist is frequently described as a two degree of freedom joint, with the largest range of motion occurring about the flexion/extension (FE) and radial/ulnar deviation (RUD) axes [1].

Wrist motion can be quantified in a variety of ways, including optical motion capture (OMC), electrogoniometry, and video analysis. OMC is widely considered to be a benchmark for kinematic measurement methods as it is accurate, non-invasive, widely used in scientific experimental studies, and does not expose participants to the radiation associated with imaging-based methods [1]. However, there are notable disadvantages to OMC. The systems are expensive, restricted to a confined laboratory setting or capture volume, and require significant time and technical expertise for the setup, operation, and data post processing [1,2]. Video capture is more widely used in ergonomic assessment, as it is more portable, and easier to setup and operate, making it practical to use in the workplace. While some experimental strategies have been successful with the extraction of continuous kinematics from video data, these strategies have not yet been widely explored for ergonomic assessment [3]. Commonly-used ergonomic assessment methodologies include rapid upper limb assessment (RULA) [4], rapid entire body assessment (REBA) [5], and strain index (SI) [6], all of which rely on expert analysis to bin postures into general ranges for subsequent interpretation.

Electrogoniometry offers a practical alternative to video analysis or OMC-based methods and has been demonstrated to have good agreement with OMC-based methods, and higher reliability than video-analysis [1,3]. Wearable ergonomic assessment tools, such as electrogoniometers, are also advantageous as they are minimally obtrusive, making them better suited for long-term monitoring in occupational settings, which could improve our understanding of chronic risk factors for occupational upper extremity injuries [7]. Early approaches used electromechanical goniometers, comprised of a rigid frame and potentiometer for angular sensing [8,9]. However, with the advent of flexible electrogoniometers, there was a shift away from using potentiometer based electromechanical goniometers. The reason for this is that flexible electrogoniometers are minimally intrusive, more robust to errors in alignment relative to the joint centers of rotation, and multiple axes of rotation can be measured with a single device [10]. Flexible electrogoniometers, however, are susceptible to crosstalk between the FE and RUD axes, necessitating algorithmic correction [11,12].

The use of inertial measurement units (IMUs) is an even more appealing approach than electrogoniometry, as they are modular, and can be placed in an even more minimally invasive manner. Results, however, have been inconsistent when applied to the calculation of wrist joint kinematics [13]. IMU performance is heavily reliant on the approach applied in the post processing of the data, and output can vary significantly depending on the underlying kinematic model used [14]. Furthermore, IMUs are affected by local ferromagnetic interference which may pose challenges in certain occupational environments [14].

Electrogoniometry and IMUs both offer the technical capability to collect joint kinematics in occupational environments; however, durability, validity, and cost of commercially available wearable systems have been highlighted as barriers to broad adoption by practicing occupational safety and health professionals [15]. In particular, average acceptable per device cost has been estimated at $72.21 (USD) which poses a significant barrier, as most valid, commercially-available devices capable of continuously monitoring joint kinematics greatly exceed this threshold [15].

Recently, our lab has developed a low-cost (<$50 (USD)) wearable device capable of recording wrist FE and RUD. The device is similar to previously used potentiometer-based electromechanical goniometers, such as the device used by Schoenmarklin and Marras in their study of hand-intensive industrial jobs [8,16], or the device used by Ryu et al. in their study of functional wrist motions [9], however, several factors make it appealing to re-explore this approach to kinematic monitoring. The low cost and high performance of modern dataloggers and miniaturized rotary position sensors make broad field implementations of electromechanical goniometers much more feasible. Additionally, as our device is 3D printed, it is trivial to customise to match participant anthropometrics which can improve device alignment with joint centers of rotation and improve accuracy. The majority of the cost associated with this device is the datalogger, which is a distinct module from the wearable transducer. This means that a single datalogger can be used repeatedly with different transducers that can be customised for diverse participant anthropometrics. This could greatly reduce the costs associated with occupational kinematic monitoring and facilitate the collection of large datasets of occupational wrist kinematics. The purpose of this study was to evaluate the accuracy of the kinematic data produced by the wearable device relative to an OMC system.

## 2. Materials and Methods

### 2.1. Device Design

**Figure 1:**
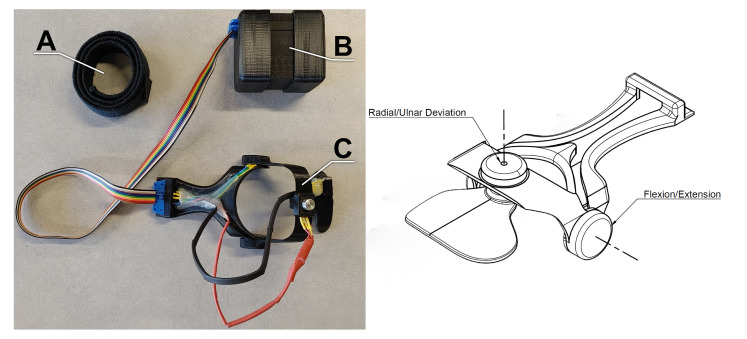
The 3D printed wearable device with wrist strap (**A**), datalogger (**B**), and transducer (**C**).

The wearable device is 3D printed in polylactic acid (PLA) and attaches to the dorsal side of the hand and forearm via double sided tape and a hook and loop wrist strap (Figure 1 ). The frame includes two hinges, instrumented with rotary position sensors, placed over the approximate anatomical centers of rotation for the FE and RUD axes of the wrist. The device can be 3D printed using a consumer grade fused filament fabrication printer and a low-cost rotary position sensor is located at each hinge point to record the corresponding joint angle.

Two wearable devices were produced based on hand and wrist anthropometric data from 50th percentile-sized male and female wrists [17]. The devices were printed in PLA and a Bourns Model 3382 rotary position sensor (Bourns, Riverside, CA, USA) was installed at each of the FE and RUD axes. Data were digitised and stored by a Teensy 3.5 microcontroller (PJRC, Sherwood, OR) in 10-bit resolution at 100 Hz.

### 2.2. Benchtop Test Procedures

**Figure 2:**
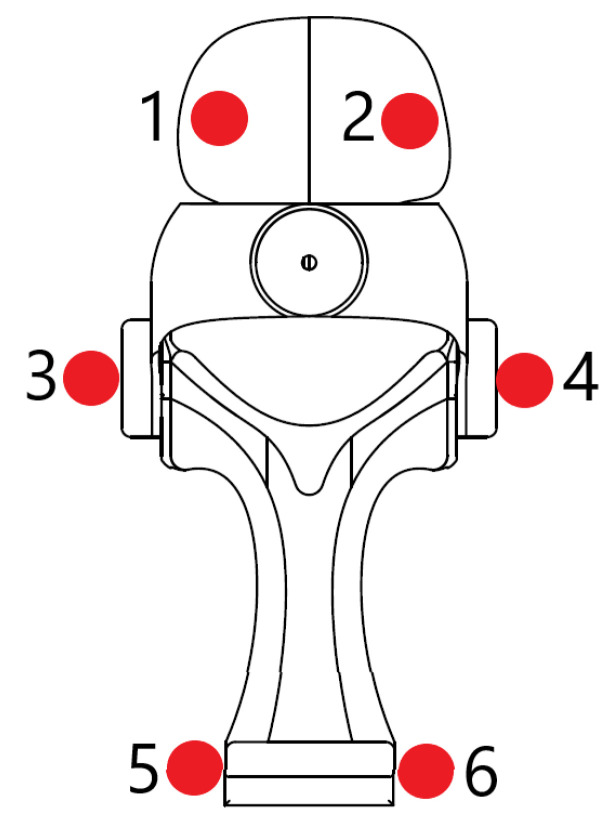
Marker locations for wearable device analysis. Markers 1 and 2 approximate the anatomical positions of the second and fifth metacarpophalangeal markers, markers 3 and 4 approximate the positions of the radial and ulnar styloid markers, and markers 5 and 6 approximate the anatomical positions of the medial and lateral humeral epicondyle markers.

To quantify baseline kinematic differences between the OMC system and wearable device, a static benchtop validation was performed, without the device being worn. All markers were placed on the wearable device, as shown in Figure 2. Note that markers 1 and 2 were placed proximally, and markers 5 and 6 were placed distally and dorsally relative to correct anatomical placement. These differences in relative marker placement when compared to correct anatomical marker placement resulted in an angular offset, which was removed by normalising to a position which would represent an approximately neutral posture if the device were being worn.

To generate a calibration, the device was manually moved through its range of motion in each axis in approximately 5° increments while holding the opposing axis approximately stationary. A linear calibration was calculated based on this data and applied to all benchtop validation data. Next, a nested set of combined FE and RUD postures were recorded. This consisted of recording 5 RUD postures (neutral posture, extreme radial deviation, extreme ulnar deviation, moderate radial deviation, and moderate ulnar deviation) at 5 different FE postures (neutral posture, extreme flexion, extreme extension, moderate flexion, and moderate extension). The wearable and OMC system were then compared using both the calibration and test data.

### 2.3. In Vivo Experimental Procedures

Ten healthy, right-hand dominant subjects (6 female and 4 male, aged 24 ± 6 years) were recruited for this study. All participants provided written informed consent and the study was approved by the University of Guelph Research Ethics Board (REB# 16-12-600).

**Figure 3:**
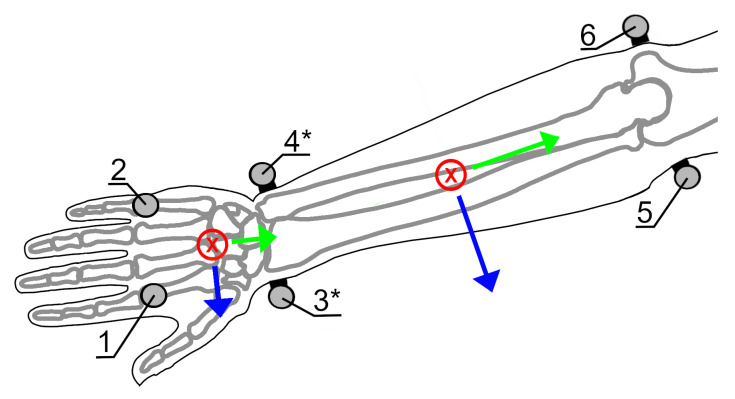
Forearm and hand coordinate systems and markerset, showing corresponding anatomical landmarks including: Second metacarpal-phalangeal joint (**1**), fifth metacarpal-phalangeal joint (**2**), radial styloid process (**3**), ulnar styloid process (**4**), medial humeral epicondyle (**5**), and lateral humeral epicondyle (**6**). * Markers 3 and 4 are placed on the wearable device.

Six, 14-mm diameter retroreflective markers were mounted on each subject’s right forearm and hand (Figure 3). OMC data were recorded by a VICON motion capture system using 5 T160 and 4 Bonita cameras (Vicon Motion Systems Ltd., Oxford, UK) with wrist FE and RUD angles recorded concurrently by the wearable device. Markers were placed on the second and fifth metacarpal-phalangeal joints, on the wearable device over the radial and ulnar styloid processes, and on the medial and lateral epicondyles of the elbow. The radial and ulnar styloid markers were placed on the wearable device in line with the flexion extension axis.

**Figure 4:**
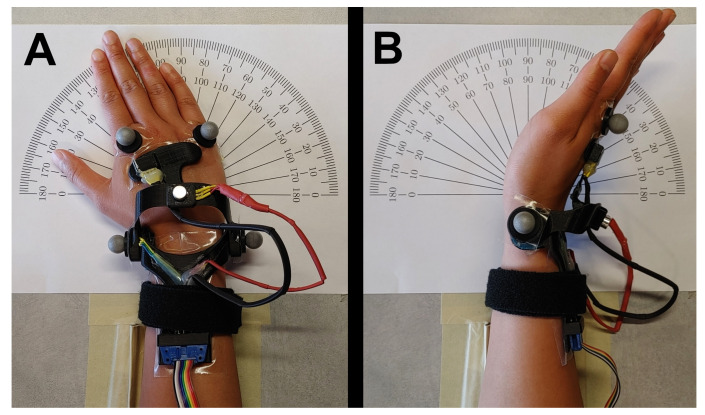
Experimental setup showing radial/ulnar deviation (**A**) and flexion/extension (**B**) measurements.

Participants performed all tasks while seated at a desk (Figure 4). First, participants performed a dynamic range of motion trial for both FE and RUD. Next, participants placed their forearm on an armrest affixed to the desk. Participants were instructed to hold static wrist angles by matching a protractor marked on the desk. Participants started at a neutral posture and increased in 10-degree increments to their maximum range of motion, then back in the same 10-degree increments to their minimum range of motion before returning to neutral posture, again by 10-degree increments. This process was performed individually for both FE and RUD angles.

### 2.4. Data Analysis

All data were analysed using a custom Python 3.9 script. Local coordinate systems based on the ISB recommendations for the forearm and hand segments were defined (Table 1) [18]. Wrist angles were calculated using Euler angles with a ZYX rotation order where the Z axis defined FE and the X axis defined RUD. Joint angles were calculated following the ISB recommendations for the wrist joint coordinate system which in turn is based on Grood and Suntay’s knee joint coordinate system [18,19]. All joint angle time series were filtered using a 4th order zero lag Butterworth low pass filter with a cut-off frequency of 10Hz. To synchronise the two data sources, the cross-correlation function was computed between the OMC and wearable data. The data were synchronised by aligning them to the global maximum of their cross-correlation function.

For each subject, a linear calibration was computed to calibrate the wearable device to the OMC system using the FE and RUD dynamic range of motion trials. This calibration was then applied to the static trial data. Performance was quantified by comparing the calibrated static trials between the OMC system and wearable.

## 3. Results

### 3.1. Benchtop Test

**Figure 5:**
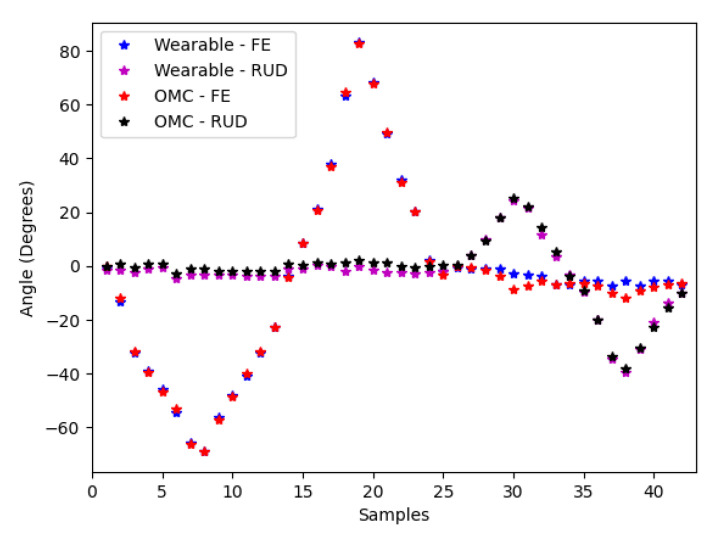
Wearable and optical motion capture (OMC) flexion/extension (FE) and radial/ulnar deviation (RUD) calibration trials, with calibration applied.

**Figure 6:**
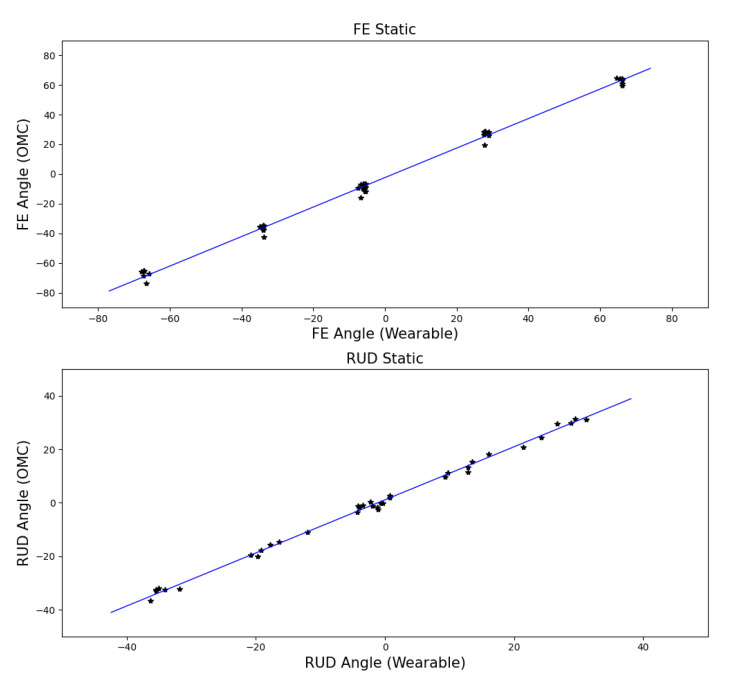
Correlation between the optical motion capture and wearable device during benchtop testing.

The calibration trial for the FE and RUD axes is shown in Figure 5 with the calibration for both FE and RUD applied. Performance was quantified using coefficients of determination which were 0.995 and 0.996 for FE and RUD respectively, as shown in Figure 6. RMSE was 3.8° and 1.7° for FE and RUD respectively.

### 3.2. In Vivo Test

**Figure 7:**
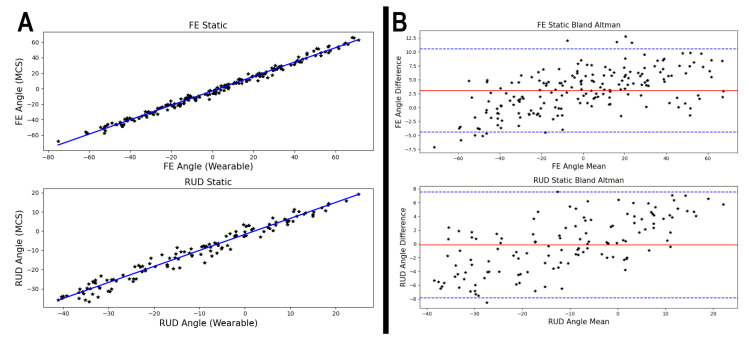
Correlation between wearable and optical motion capture (OMC) for flexion/extension (FE) and radial/ulnar deviation (RUD) (**A**) mean-difference plots quantifying agreement between the wearable and OMC for FE and RUD (**B**). Each point represents one posture.

Individual and cumulative performance of the wearable device relative to the OMC system are presented in Table 2. Correlation between systems is quantified by the coefficient of determination and depicted graphically in Figure 7. Measurement error is presented as the root mean squared error over all postures expressed by participant and FE as well as RUD.

Correlation was high across all participants with a minimum $R^{2}$ of 0.973. Agreement between the two systems was quantified using Bland–Altman analysis [20]. Mean differences between the systems were 3.1° and −0.2° with limits of agreement of ±7.4° and ±7.7° for FE and RUD, respectively (Figure 3).

## 4. Discussion

The aim of this study was to quantify the performance of a low-cost 3D printed wearable device relative to an OMC system for measuring wrist FE and RUD angles. Overall, the wearable device performed quite well relative to the OMC system with high correlation and low bias. The discrepancy between the two systems was more evident when examining the RMSE and 95% limits of agreement, however, this still represents a major improvement in data resolution relative to video analysis commonly used for ergonomic assessment. Video-based ergonomic assessment, while valid, suffers from uncertainty due to the coarse resolution caused by dividing the range of motion into bins, typically 3 to 6, as well as susceptibility to parallax errors caused by suboptimal camera placement [21].

Our device produces results comparable to recent findings for flexible electrogoniometers. McHugh et al. found biases of −5.2° and −0.8 with 95% limits of agreement of ±9.1° and ±3.3° for FE and RUD, respectively when using a strain gauge-based electrogoniometer [1]. Performance of our wrist wearable was similar despite the relative simplicity and low cost of our device compared to the strain gauge-based or fiber optic electrogoniometers frequently used for kinematic data collection.

Recently, interest has largely been focused on the use of inertial measurement units (IMUs), as they are unobtrusive, and can produce results comparable with OMC, however their performance for measuring wrist angles has been varied. In their recent review of kinematic analysis using IMUs, Poitras et al. found reported RMSE ranging from 3°–20° and 3°–30° for FE and RUD, respectively [13]. It should be noted that performance for state of the art commercially available IMU systems is on the favourable end of that range. In their evaluation of the Xsens MVN system, Robert-Lachaine et al. found a RMSE of 3.8° and 3.6° with biases of −1.0° and −1.3° with limits of agreement of ±6.9° and ±5.9° [14]. Our results compare favourably especially when considering that the type of IMU system used was more similar to an OMC system in terms of cost and technical expertise required, than this simple joint specific monitoring tool.

The benchtop testing serves an important purpose in quantifying the inherent differences in the way that the wearable and OMC system measure kinematics. Applying anatomically-based coordinate systems to calculate angular data for this benchtop test resulted in crosstalk. This is evident at samples 30 and 38 in Figure 5, where the OMC derived FE angle decreases at a high RUD angle. This phenomenon arises from the fact that the wearable device and OMC system define the center of rotation for RUD at different points. Wrist joint motion is particularly complex, and there is no consensus for the precise determination of the FE and RUD rotation axes in vivo [22]. This means that a generalised OMC implementation, such as the one used here, will always suffer from some crosstalk, and thus falls somewhat short of being a true gold standard. While it would be possible to redefine the OMC coordinate systems for the hand and forearm such that they precisely match the wearable device, and minimise reported error, our goal was to evaluate the performance of the wearable device relative to a conventional application of OMC. Here we have mitigated the effects of FE and RUD axis mismatch by evaluating the device for planar motion in FE and RUD, separately. Unsurprisingly both RMSE and $R^{2}$ for the benchtop test case outperform the cumulative RMSE and $R^{2}$ for our in vivo study. However, benchtop test results still remain within the performance range observed during the in vivo experiment.

This study is narrow in scope, focusing on quantifying the accuracy of electromechanical goniometry relative to current practices for OMC. The accuracy found here, combined with the historical validity of this style of instrument make a strong argument for deploying the device to study ergonomic risk factors. Within the study of ergonomics, there has been interest in developing risk assessments which leverage the availability of wearable technologies. A shift towards wearable kinematic monitoring would allow for more comprehensive monitoring of ergonomic risk factors [23]. This would also allow an exploration of individual-specific, as well as job-specific postural risk factors [16]. Furthermore, widespread application of wearable devices could increase the volume, resolution, and reliability of the available kinematic data when compared to conventional video analysis methods [3]. The availability of low-cost potentiometer-based rotary position sensors such as the Bourns Model 3382, combined with the design flexibility afforded by desktop 3D printing provide a compelling opportunity to produce wearable devices for occupational assessment.

## Figures and Tables

**Table 1:** Definitions of the forearm and hand coordinate systems used to calculate wrist angles, orange markers indicate calculated midpoints, X-axes point volarly.

Y-Axis	X-Axis	Z-Axis
Forearm		
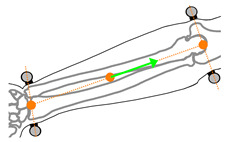 Vector between the midpoint of the radial and ulnar styloid processes and the midpoint of the medial and lateral epicondyles of the elbow	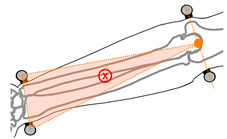 Vector normal to the plane formed by the radial and ulnar styloid processes and the midpoint of the medial and lateral epicondyles of the elbow	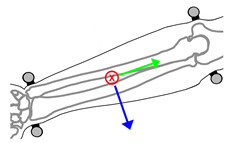 Cross product of the X-axis of the forearm and Y-axis of the forearm
Hand		
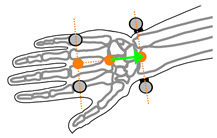 Vector between the midpoint of radial and ulnar styloid processes, and the midpoint of the second and fifth metacarpal-phalangeal joints	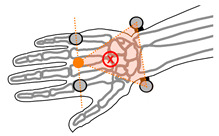 Vector normal to the plane formed by the radial and ulnar styloid processes and the midpoint of the second and fifth metacarpal-phalangeal joints	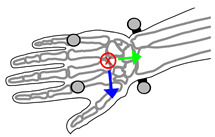 Cross product of the X-axis of the hand and Y-axis of the hand

**Table 2:** Wearable device performance relative to OMC for all subjects. Root mean squared error (RMSE) and the coefficient of determination (R${}^{2}$) are presented for flexion/extension and radial/ulnar deviation. All R${}^{2}$ are significant (*p* < 0.0001). Cumulative performance is calculated across the entire dataset.

Participant	Flexion/Extension		Radial/Ulnar Deviation	
	*R* ${}^{2}$	RMSE (°)	*R* ${}^{2}$	RMSE (°)
Participant 1	0.998	3.0	0.973	3.9
Participant 2	0.995	3.0	0.976	3.4
Participant 3	0.991	7.4	0.980	4.6
Participant 4	0.986	5.2	0.992	4.5
Participant 5	0.994	4.6	0.992	4.5
Participant 6	0.992	6.2	0.975	5.3
Participant 7	0.992	5.7	0.987	1.9
Participant 8	0.994	3.6	0.996	5.3
Participant 9	0.997	5.5	0.995	2.9
Participant 10	0.998	5.0	0.990	2.0
Cumulative Performance	0.991	4.7	0.966	3.9

## Data Availability

Access to data is restricted due to restrictions placed by the University of Guelph Research Ethics Board to protect the privacy of the study participants.

## Abbreviations

The following abbreviations are used in this manuscript:

FE	Flexion/extension
RUD	Radial/ulnar deviation
OMC	Optical motion capture
IMU	Inertial measurement unit
